# Evaluation of age group and sex differences in the measurement of patellar height of pediatric knee in a Korean population

**DOI:** 10.3389/fped.2022.1021147

**Published:** 2023-01-16

**Authors:** Yoon Hae Kwak, Soo-Sung Park, Aaron J. Huser, Hyo Won Lim, Sharkawy wagih Abdel Baki, Yong-Gon Koh, Ji-Hoon Nam, Kyoung-Tak Kang

**Affiliations:** ^1^ Department of Orthopedic Surgery, Asan Medical Center, Seoul, South Korea; ^2^ Paley Advanced Limb Lengthening Institute, St. Mary's Hospital, West Palm Beach, FL, United States; ^3^ Department of Orthopedic Surgery, Aswan University Hospital, Aswan, Egypt; ^4^ Department of Orthopaedis Surgery, Severance Children's Hospital, Seoul, South Korea; ^5^ Joint Reconstruction Center, Department of Orthopaedic Surgery, Yonsei Sarang Hospital, Seoul, South Korea; ^6^ Department of Mechanical Engineering, Yonsei University, Seoul, South Korea

**Keywords:** patella height, radiograph, Korean pediatric patients, reliability, insall-Salvati index

## Abstract

**Introduction:**

Various methods based on bony landmarks are used to determine patellar height. This study analyzed five methods for patellar height measurement on lateral knee radiographs, namely, the Insall–Salvati, Koshino–Sugimoto, Blackburne–Peel, modified Insall–Salvati, and Caton–Deschamps methods.

**Methods:**

Overall, 425 pediatric participants (221 males, 204 females; age range 5–18 years) were included and were divided equally into three age groups (A, 5–10 years; B, 11–13 years; and C, 13–18 years). For the comparison of the applicability of each method, the applicable probabilities for each age group and sex-based differences were analyzed using logistic regression techniques. Intra-rater reliability and inter-rater variability were analyzed by two trained raters.

**Results:**

The Koshino–Sugimoto method was applicable to all patients. The 80% applicable age of female patients was lower than that of male patients for the Blackburne–Peel (male = 11.9, female = 11) and Caton–Deschamps (male = 11.9, female = 11.1) methods. However, in the Insall–Salvati (male = 12, female = 12.1) and modified Insall–Salvati (male = 12.6, female = 13.1) methods, the 80% applicable age in male patients was lower than that in female patients. The Koshino–Sugimoto method showed the highest variability in group B, while the Insall–Salvati showed the highest variability in group C. In terms of intra-observer reliability, the Caton–Deschamps method showed the same reliability as the Insall–Salvati method, in group C.

**Conclusions:**

Our results demonstrated differences in the reliability, variability, and applicability of patellar height measurement methods according to age group. The applicability of patellar height measurement methods also differed according to sex. Therefore, based on age group and sex, different methods should be used for patellar height measurement in pediatric patients.

## Introduction

The patella is an important component of the extensor mechanism in the knee joint (1, 2). Evaluation of high- and low-riding patella (alta and baja, respectively) has been used for the analysis of patello-femoral (PF) instability and PF joint pain (3). Additionally, patella alta is a well-known anatomic risk factor for PF joint pain (4, 5).

Understanding patellofemoral morphology and its relationship to maltracking is important in patellofemoral pain in 7 to 29% of adolescents (6). So, in patients with patellar instability or patellofemoral pain, a measurement of patellar height should be considered in the workup for treatment (7).

Various indices have been defined for measuring the height of the patella on lateral knee radiographs (8–11). These methods have both positive and negative aspects. The most commonly used methods are the Insall–Salvati (IS), Koshino–Sugimoto (KS), Blackburne–Peel (BP), modified IS (modIS), and Caton–Deschamps (CD) methods (8, 12, 13). However, most of these indices rely on bony landmarks; thus, their application in pediatric population, whose skeletons are not yet fully mature, is unjustified (14). The application of magnetic resonance imaging (MRI) for the analysis of patellar height has reportedly provided reliable patellar height length (14, 15). MRI may also prove to be an additional modality for the accurate diagnosis of patellar height, while avoiding unnecessary radiation. However, performing MRI to measure patellar height in all pediatric patients is difficult. MRI is less cost-effective than radiography and sedation may be required for pediatric patients (16).

Moreover, a recent study showed that MRI can overestimate patellar height compared to lateral knee radiographs and recommended conventional radiography (CR) as the preferred method for measuring patellar height (17). Additionally, a previous study showed that the BP and KS methods were reliable for the assessment of lateral knee radiographs in pediatric patients (8, 14, 18). Another study reported the best inter-observer agreement was with the CD method on lateral knee radiographs (19). The CD method is a simple, reliable, and reproducible index that is not affected by skeletal maturation (19). Park et al. assessed which of the three methods, among IS, BP, and KS, was most appropriate for measuring patellar height in pediatric patients; however, they did not consider modIS and CD methods (14). Recently, Kurowecki et al. reported that IS and patella alta, as determined on MRI, were comparable to those determined on radiography in pediatric patients (20). However, these two studies on pediatric patients did not consider other methods to measure patellar height and had limitations of modest sample sizes (14, 20). Moreover, while many previous studies have evaluated the reliability and variability of various patellar height measurement methods (7, 21–23), data on pediatric patients are scarce. To the best of our knowledge, studies reporting on the reliability and variability of patellar height by widely used methods based on lateral knee radiography in a large number of pediatric patients are absent.

Therefore, the present study aimed to evaluate the inter-observer and intra-observer reliabilities of five patellar height measurement methods (the IS, KS, modIS, BP, and CD) in the assessment of lateral knee radiographs in pediatric patients. This study also evaluated which measurement methods were most applicable to specific sex and age groups. We hypothesized, that different methods for patellar height measurement should be used according to age group and sex, in pediatric patients.

## Material and methods

This study is about a Korean population that has a sedentary lifestyle, uncommon in other ethnicities. This retrospective study analyzed records from the radiology department to obtain details of pediatric patients aged 5–18 years with previous lateral knee radiographs, after obtaining institutional review board approval. The exclusion criteria included: evidence of PF disorders (pain, cartilage lesions or arthritis), multi-ligament injuries, osteoarthritis, bone fractures, patella magna, bipartite patella, previous knee surgeries, and limb deformities (24). We also excluded radiographs those excessively diverging from 30° flexion or rotation from true lateral view. This study included a total of 425 patients (425 knees; 221 male and 204 female). The patients were divided into three age groups (A, 5–10 years; B, 11–13 years; and C, 13–18 years) (14, 25, 26). The mean patient age was 12 years (standard deviation, 3.5; range, 5.0–18 years) (Table 1).

**Table 1:** Comparison of the age and sex ratio among age groups.

Parameter	Total (*n* = 425)	Group A (*n* = 144)	Group B (*n* = 133)	Group C (*n* = 148)
	Mean ± SD (range)	Mean ± SD (range)	Mean ± SD (range)	Mean ± SD (range)
Age	12.0 ± 3.6 (5,18)	7.9 ± 1.6 (5,10)	12.0 ± 0.8 (11,13)	15.9 ± 1.5 (14,18)
Male/female	221/204	67/77	78/55	76/72

**Figure 1:**
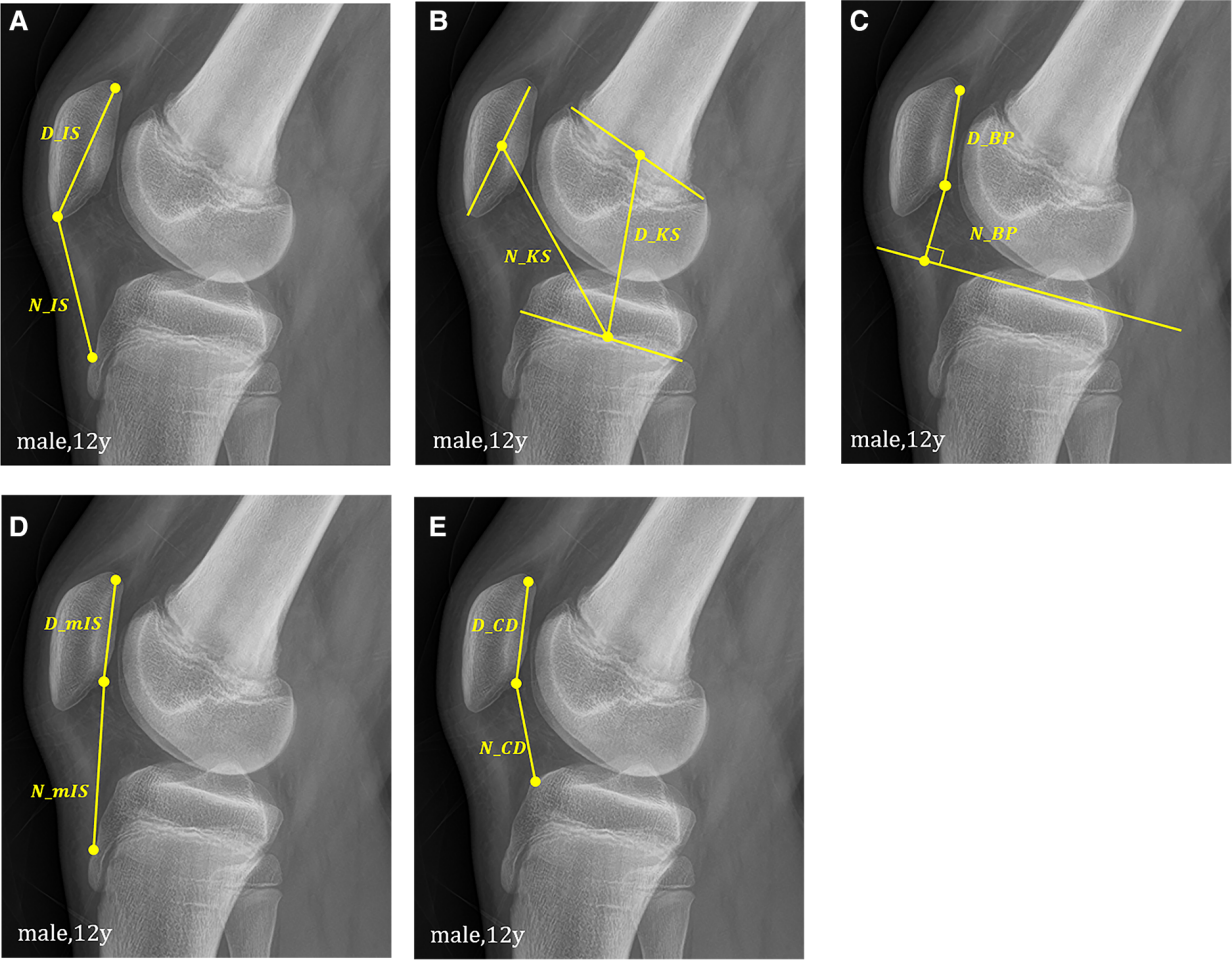
Schematic representation of (**A**) Insall-Salvati, (**B**) Koshino-Sugimoto, (**C**) Blackburne-Peel, (**D**) modified Insall-Salvati, (**E**) Caton-Deschamps method.

Radiographs were obtained on a GE Definium 8,000 instrument. For the lateral view, the patients were positioned with the knee at 30° of flexion in lateral recumbent position of the measuring side to obtain a true lateral view with overlapping posterior condyles. The five methods used for the measurement of patellar height index were IS, KS, BP, modIS, and CD. The IS method calculates the index as the ratio of the patella tendon length (N_IS) to the patella bone length (D_IS) (Figure 1A). In Figure 1, D and N indicate the denominator and numerator, respectively. This convention was applied to all other methods. The KS method calculates the index as the ratio of the distance between the center of the patella and the center of the proximal tibial physis (N_KS) to the distance between the center of the femoral distal physis and center of the proximal tibial physis (D_KS) (Figure 1B). The BP method calculates the index as the length of a perpendicular line from the tibial plateau to the inferior point of the patellar articular surface (N_BP) and the length of the patellar articular surface (D_BP) (Figure 1C). The modIS method calculates the index as the ratio of the distance from the inferior point of the patellar articular surface to the patellar tendon attachment on the tibia (N_mIS) to the length of the patellar articular surface (D_mIS) (Figure 1D). The CD method calculates the index as the ratio of the distance from the inferior point of the patellar articular surface to the anterior most point of the tibial plateau (N_CD) to the length of the patellar articular surface (D_CD) (Figure 1E). All indices were measured on lateral radiographs.

Some methods were not applicable, depending on the patient age, because the participants were pediatric patients with incomplete ossification. Applicability was defined as the possibility to identify the bone landmarks referenced by each measurement. For the comparison of the applicability of each method, the applicable probabilities were calculated for each age group. Sex-based differences were also analyzed. The intra-rater reliability and inter-rater variability were analyzed by two trained raters. The raters were selected from the authors. For the analysis of reliability and variability, all patients and methods were used without sampling. The two measurements were taken at least 2 weeks apart.

### Statistical analysis

**Figure 2:**
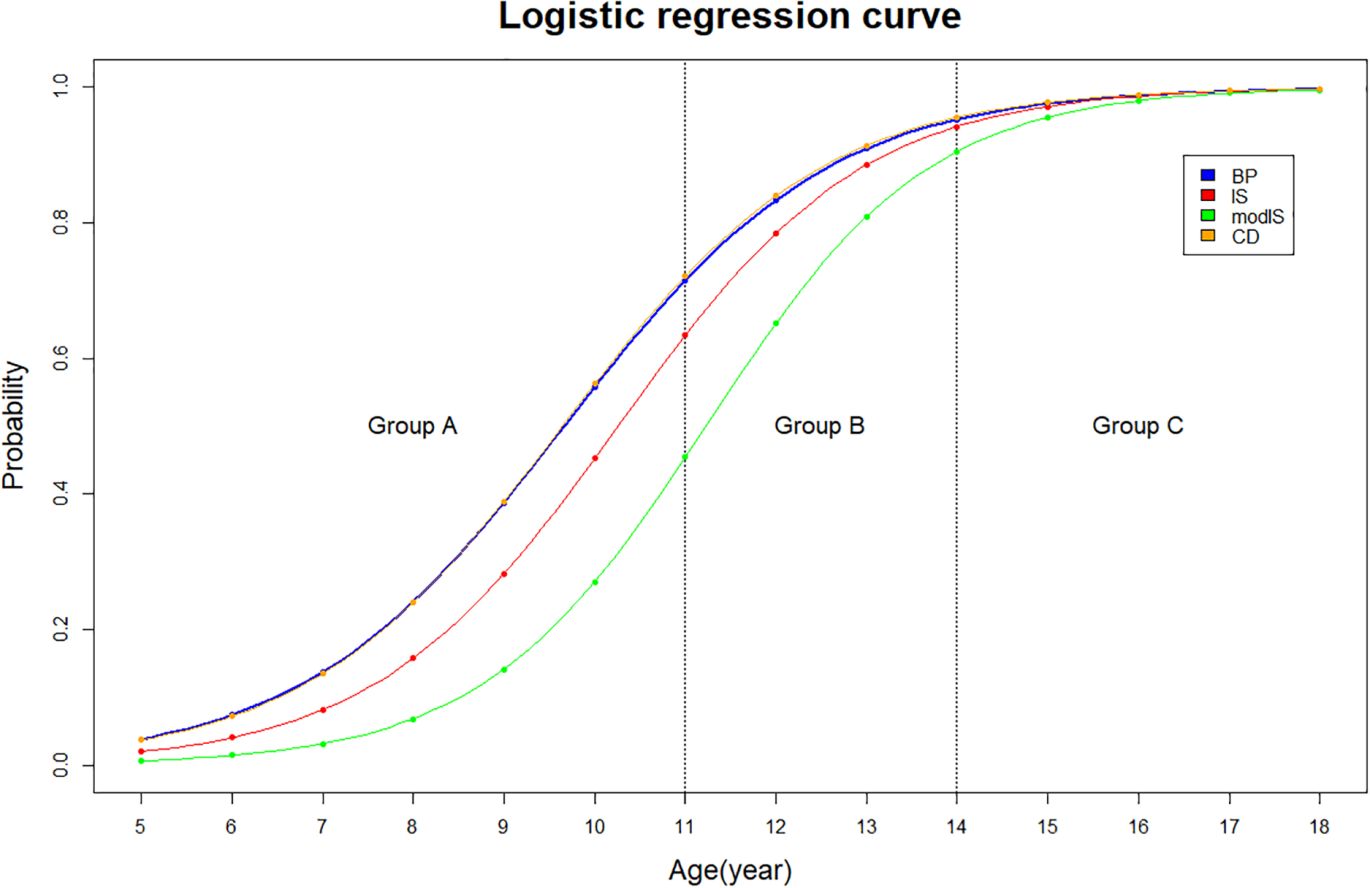
Logistic regression curve of the applicable probability regarding each measurement method.

**Figure 3:**
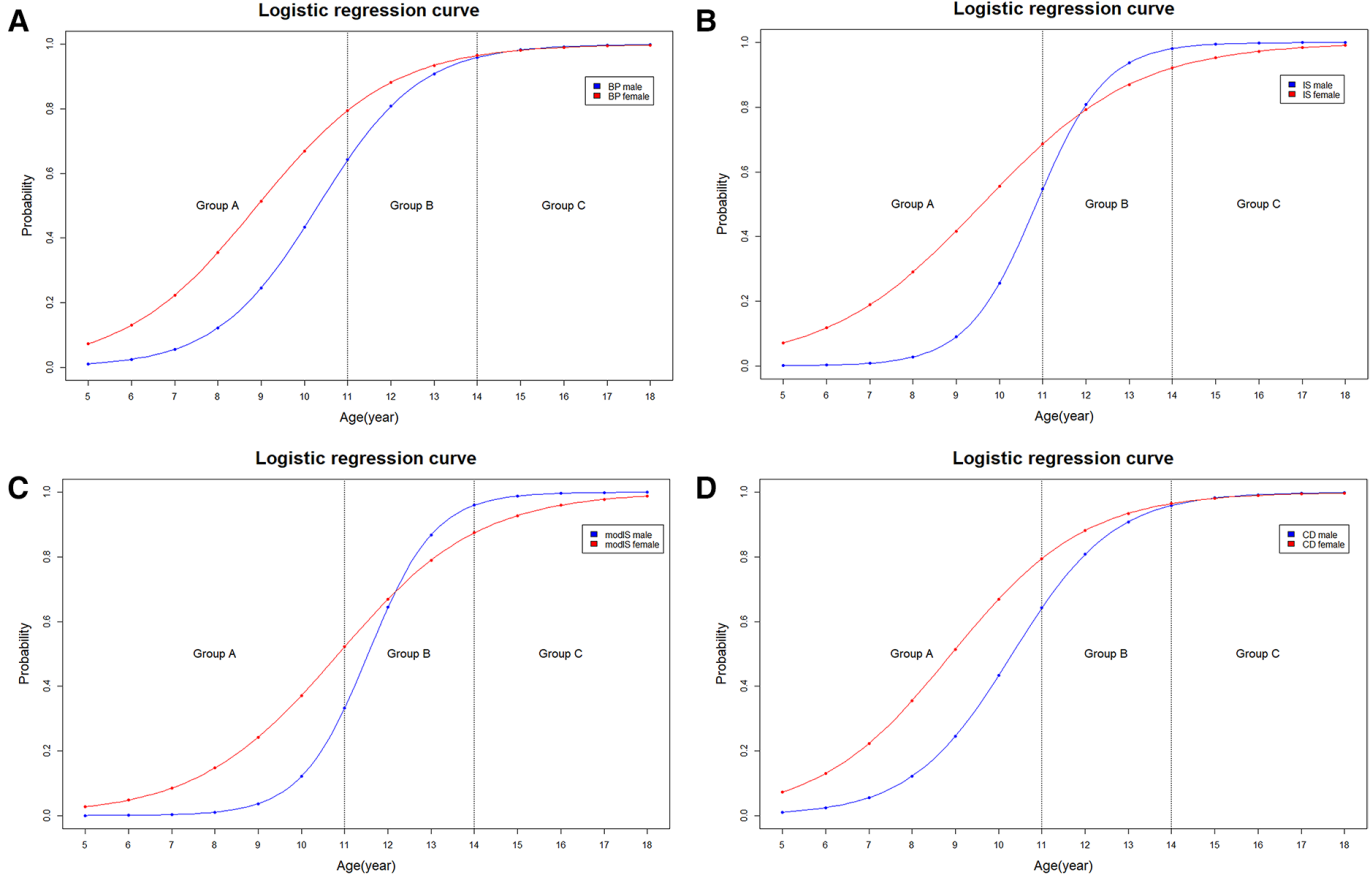
Logistic regression curve of the applicable probability between Male and female regarding (**A**) Blackburne-Peel, (**B**) Insall-Salvati, (**C**) modified Insall-Salvati, (**D**) Caton-Deschamps method.

The arithmetic mean and standard deviation values were calculated to describe the morphological data in each age group (A, B, and C). The male and female ratio was calculated (Table 1). The statistical data were analyzed using PASW Statistics for Windows, version 18.0 (SPSS, Inc., Chicago, IL, USA). For the applicability evaluation, logistic regression was conducted using the applicable probability for each age group and sex (Figures 2, 3). Intra-class correlation coefficients (ICCs) were used to evaluate the intra- and inter-rater reliabilities.

## Results

The applicability of the KS method was 100% in all groups. In group A, the applicability of the BP, IS, modIS, and CD methods were 31.9%, 18%, 11.8%, and 31.9%, respectively (BP = CD > IS > modIS). Figure 1F show that the patellar articular cartilage and tibial tuberosity cannot be identified in group A. In group B, the applicability of the BP, IS, modIS, and CD methods were 75.9%, 78.8%, 60.9%, and 75.9%, respectively. In group C, the applicability of all methods was >97%. The binomial logistic regression curve is shown in (Figures 2, 3). In the curve, the y-axis shows the possibility of measurement at each age. The BP and CD methods showed the lowest ages at 80% applicability.

In male patients in group A, the applicability of the BP, IS, modIS, and CD methods was 20.8%, 7.4%, 2.9%, and 20.8%, respectively, and in female patients, it was 41.5%, 27.2%, 19.4%, and 41.5%, respectively. The applicability for female patients was higher than that for male patients in group A. However, the modIS method in group B showed a slightly higher applicability in male patients than in female patients (61.5% vs. 60.0%). A binomial logistic regression curve was drawn (Figure 3). The 80% applicable age of female patients was lower than that of male patients in the BP and CD methods. However, the 80% applicable age was lower in male patients for the IS and modIS methods.

The inter-observer variability was highest for the KS method (0.90) and lowest for the IS method (0.30) in group B (Table 2). The inter-observer reliability tended to increase with age for the IS method and was lowest for the KS method in group C (Table 2) The intra-observer reliability was highest for the KS method and lowest for the BP method in group B (Table 3). The intra-observer reliability of the IS and CD methods tended to increase with age.

**Table 2:** Inter-observer variability of patellar height ratios on x-ray.

Group	Observers	IS		BP		CD		modIS		KS	
		ICC	95% CI	ICC	95% CI	ICC	95% CI	ICC	95% CI	ICC	95% CI
A	First	NA	NA	NA	NA	NA	NA	NA	NA	0.85	0.8∼0.89
	Second	NA	NA	NA	NA	NA	NA	NA	NA	0.95	0.93–0.96
	Overall	NA	NA	NA	NA	NA	NA	NA	NA	0.90	0.87∼0.92
B	First	0.25	−0.1∼0.56	0.61	0.11∼0.82	0.56	0.41∼0.68	0.40	0.18∼0.58	0.94	0.92–0.95
	Second	0.42	0.16∼0.6	0.66	0.53∼0.76	0.64	0.5∼0.74	0.33	0.09∼0.53	0.86	0.74∼0.92
	Overall	0.3	−0.03∼0.54	0.64	0.5∼0.74	0.61	0.51∼0.69	0.36	0.17∼0.52	0.90	0.87∼0.92
C	First	0.52	−0.01∼0.77	0.69	0.45∼0.81	0.70	0.61∼0.77	0.58	0.46∼0.68	0.74	0.54∼.084
	Second	0.92	0.89∼0.94	0.49	0.23∼0.66	0.61	0.47∼0.71	0.57	0.44∼0.67	0.40	0.06∼0.62
	Overall	0.67	0.5∼0.77	0.57	0.49∼0.64	0.65	0.57∼0.72	0.57	0.49∼0.64	0.52	0.23∼0.68

**Table 3:** Intra-observer reliability of patellar height ratios on x-ray.

Group	Observers	IS		BP		CD		modIS		KS	
		ICC	95% CI	ICC	95% CI	ICC	95% CI	ICC	95% CI	ICC	95% CI
A	First	NA	NA	NA	NA	NA	NA	NA	NA	0.93	0.9–0.95
	Second	NA	NA	NA	NA	NA	NA	NA	NA	0.90	0.86–0.93
	Overall	NA	NA	NA	NA	NA	NA	NA	NA	0.91	0.89–0.93
B	First	0.48	0.07∼0.71	0.51	0.05∼0.74	0.76	0.66∼0.83	0.73	0.59∼0.82	0.91	0.87–0.93
	Second	0.86	0.79∼0.9	0.76	0.67∼0.83	0.61	0.47∼0.72	0.63	0.48∼0.75	0.93	0.87–0.96
	Overall	0.66	0.51∼0.75	0.65	0.45∼0.76	0.68	0.6∼0.75	0.71	0.63∼0.78	0.92	0.90–0.94
C	First	0.59	0.12∼0.79	0.35	-0.04∼0.6	0.70	0.6∼0.77	0.64	0.52∼0.74	0.72	0.34–0.86
	Second	0.93	0.68∼0.97	0.87	0.82∼0.9	0.72	0.63∼0.79	0.67	0.57∼0.76	0.59	0.47–0.68
	Overall	0.71	0.63∼0.78	0.53	0.3∼0.67	0.71	0.65∼0.76	0.65	0.58∼0.71	0.66	0.59–0.72

The inter-observer variability was lower than the intra-observer reliability. The intra-observer reliability was highest for the IS and CD methods and lowest for the modIS method in group C (Table 3).

## Discussion

The most important finding of this study was that the measurement of patellar height in pediatric patients required different methods according to age group and sex. In pediatric patients, ossification is incomplete; thus, our raters found that only the KS method could be used to measure patellar height in patients <11 years of age. The applicability in female patients was higher than that in male patients in group A, indicating that the radiographic features necessary to make the measurements appears at a younger age in females as compared to males.

Depending on previous studies about patellar height measurements in pediatric age group, there was several age groupings. Our references were the studied by Park MS (14) et al. and Beck JJ et al. (25). Our grouping is based on patella growth and ossification. Pennock AT et al. (26) recently studied that patella completed ossification at median age of 11.9 years in females and 13.7 years in males. So, we used 3 age groups 11–13 years old as one group and younger age as one and older age as one group.

Our results show a 100% applicability of the KS method in all age groups, consistent with previous reports (14). The KS method is the only method to use the distance between the distal femoral physis and proximal tibial physis. It does not rely on the further ossification of the tibial tuberosity or tibial plateau like the other methods. This is the likely why the KS method was 100% applicable in this study. An applicability of >80% for the BP, IS, modIS, and CD methods was found at ages 11.6, 12.1, 12.9, and 11.6 years, respectively. The BP and CD methods use the tibial plateau line as part of the numerator, the IS uses the length of the tendon and modIS methods uses tibial tuberosity. For the denominators the BP, CD and modIS use the patellar articular surface and the IS uses the length of the patella. These differences in the >80% applicability age are likely represented by the choice of the numerator and demonstrate that the development of the ossific nuclei for sufficient measurement using the tibial plateau occurs 6–12 months earlier than the patellar tendon length and tibial tuberosity. Interestingly, the length of the patellar tendon is likely based on the ossification of the tibial tuberosity and at some point the ossification of the tuberosity is sufficient enough to measure patellar tendon length, but not enough to mark the proximal end.

MRI visualizes cartilaginous structures, so there are previous studies related to MRI and radiographs in the pediatric knee (14, 20). Kurowecki et al. showed Insall–Salvati ratio derived from MRI and radiographs in children showed strong association and Park et al. suggested to apply the IS in patients older than 13 years with complete ossification and the KS in patients with incomplete ossification. Still there is limited to no data available on modalities other than radiography in this population or, when present, it is often reported combined with data from adults, so further study should be considered to compare between MRI and radiographs in skeletally immature patients.

In the BP and CD methods, the 80% applicable age was lower in female patients. In contrast, the 80% applicable age was lower in male patients when the IS and modIS methods were used. This could be indicate that the tibial plateau line is sufficiently ossified earlier in female patients and tibial tuberosity landmark in male patients. This finding agrees with the previous work done on this topic that the stage of skeletal maturity of bony landmarks determines the applicability of methods at particular ages (14).

Intra-observer reliability and inter-observer variability varied according to the age group for each method. The KS method was the only method that could be assessed in group A, and had excellent (≥.90) reliability and variability scores indicating it is an excellent method for this age group. Again, this is likely due to the use of the physes for measurement. This was also true for group B with ≥.90 ICC. This information is consistent with previous reports in pediatric patients (14) and is the recommended measurement method for patients under the age of 13 years.

For group C, the IS method demonstrated the best inter-observer and intra-observer reliabilities. This trend is the same as that reported previously (22, 23, 27). The moderate to good reliability and variability of the IS method might be explained by familiarity of the raters with this measurement and ease of identification of the landmarks necessary to perform the calculation after the age of 13. This agrees with the findings in antecedent reports (23). The difference noted in the intra-observer variability for the IS method in group B from poor (ICC. 30) to moderate in group C (ICC. 67) demonstrate this point.

The CD method demonstrates moderate reliability and variability in group C. Previous reports have suggested that bony landmarks used in this method are easily identifiable and reproducible (19). The IS, CD and modIS methods all demonstrate similar reliability and variability in children >13 years of age and can be used for measurement patellar height on radiographs. The BP method had poor to moderate reliability and moderate variability in group C and this study suggests that the aforementioned methods are more suitable for a consistent measurement.

Lastly, the KS method had moderate reliability and poor to moderate variability in the older age group. As discussed previously, this method uses the growth plates and may be more difficult in patients whose physes have closed.

This study has three limitations. First, the degree of knee flexion and rotation from the true lateral could not be controlled, owing to the retrospective nature of the study (14). Further study using three dimensional computed tomography should be considered to control the rotation. Second, a lack of ethnic diversity was present in the study population; thus, the results may not be generalizable to other populations. Third, the study focused on the reliability and variability of five methods for measuring patellar height; hence, the findings are not directly applicable to the surgical outcomes.

However, to the best of our knowledge, this study included the largest reported number of pediatrics to examine the reliability and variability of five methods for patellar height measurements. We also investigated the applicability of these methods according to sex and age group.

## Conclusions

Among the five patellar height measurement methods, the KS method was most reliable in groups A and B, while the IS method was ideal for group C, although the CD and modIS method was also reliable. The reliability, variability and applicability differed according to the patellar height measurement method for each age group. The applicability of the patellar height measurement methods also differed according to sex. Therefore, different methods for measuring patellar height should be used according to age group and sex in pediatric patients.

## Data Availability

The original contributions presented in the study are included in the article/Supplementary Material, further inquiries can be directed to the corresponding author/s.
